# *Clonorchis sinensis* acetoacetyl-CoA thiolase: identification and characterization of its potential role in surviving in the bile duct

**DOI:** 10.1186/s13071-015-0728-2

**Published:** 2015-02-25

**Authors:** Jinsi Lin, Hongling Qu, Guishan Chen, Lei He, Yanquan Xu, Zhizhi Xie, Mengyu Ren, Jiufeng Sun, Shan Li, Wenjun Chen, Xueqing Chen, Xiaoyun Wang, Xuerong Li, Chi Liang, Yan Huang, Xinbing Yu

**Affiliations:** Department of Parasitology, Zhongshan School of Medicine, Sun Yat-sen University, 74 Zhongshan 2nd Road, Guangzhou, 510080 People’s Republic of China; Key Laboratory for Tropical Diseases Control, Ministry of Education, Sun Yat-sen University, Guangzhou, 510080 People’s Republic of China

**Keywords:** *Clonorchis sinensis*, Acetoacetyl-CoA thiolase, Enzymatic activity, Survival rate, Lecithin, Hypercholesteremia

## Abstract

**Background:**

*Clonorchis sinensis* (*C. sinensis*) inhabits in bile duct of the host. However, the mechanisms involved in why *C. sinensis* can survive in the bile environment containing lipids have not yet been explored. In this study, *C. sinensis* acetoacetyl-CoA thiolase (*Cs*ACAT), a member of the thiolase family which has a key role in the beta oxidation pathway of fatty acid production, was identified and characterized to understand its potential role in adapting to the bile environment.

**Methods:**

The encoding sequence, conserved domains and spatial structure of *Cs*ACAT were identified and analyzed by bioinformatic tools. Recombinant *Cs*ACAT (r*Cs*ACAT) was obtained using a procaryotic expression system. The expression pattern of *Cs*ACAT was confirmed by quantitative real-time PCR, western blotting, and immunofluorescence. Gradients of lecithin were then set to culture *C. sinensis* adults *in vitro* and the survival rate of *C. sinensis* was analyzed, as well as the expression level and enzymatic activity of *Cs*ACAT in different lipid environments. Hypercholesteremia rabbit models were established by feeding with a hyperlipidemic diet and then infected intragastrically with *C. sinensis*. One and a half months later, the worm burdens and the expression level of *Cs*ACAT was detected.

**Results:**

*Cs*ACAT was confirmed to be a member of the thiolase family and present in the excretory/secretory proteins of *C. sinensis. Cs*ACAT was specifically localized at the vitellarium and sub-tegumental muscle layer in adult worms. The mRNA level of *Cs*ACAT in eggs was higher than those in adult worms and metacercariae. When adult worms were cultured with higher concentration of lecithin, the expression level and enzyme activity of *Cs*ACAT were up-regulated. The survival rate of adult worms was higher than control group. More adult worms were recovered from hypercholesteremia rabbit models. The expression level of *Cs*ACAT in these worms was higher than control group.

**Conclusions:**

Our results implied that *C. sinensis* might sense lipid levels and survive better in the bile environment with higher lipid levels. *C. sinensis* might modulate the expression and enzymatic activity of *Cs*ACAT, an enzyme involved in fatty acid metabolism, for energy or physical requirements to adapt to the host.

**Electronic supplementary material:**

The online version of this article (doi:10.1186/s13071-015-0728-2) contains supplementary material, which is available to authorized users.

## Background

*Clonorchis sinensis* (*C. sinensis*), a human liver fluke, is one of the major food-borne parasites in China [1,2]. 15–20 millions people are infected with *C. sinensis* and 1.52 millions show symptoms or complications mainly in China, Korea, East Russia, and Vietnam [1]. *C. sinensis* infection is thought to be a significant risk factor of cholangiocarcinoma and hepatocellular carcinoma in humans [3-5], and thus is included in control programs of neglected tropical diseases by WHO [2].

To efficiently adapt to the host, a parasite mainly changes its metabolism and morphology throughout a complex life cycle [6-8]. *C. sinensis* lives in the bile environment which is mainly made up of water, lecithin, cholesterol, fatty acids and bile salts [9,10]. All enzymes involved in the pathway of fatty acid beta oxidation moderately expressed in the transcriptome of *C. sinensis* adults [11], while they were not integrally expressed in blood fluke of human, *Schistosoma japonicum* (*S. japonicum*) [12]. It was hinted that these two types of parasites adopted a different metabolic mode of fatty acid production in different living environments. Their main sources of energy might be also different from each other. The mechanisms involved in why *C. sinensis* can survive in the bile environment containing relatively higher lipid have not yet been elucidated.

Eukaryotic thiolases have key roles in many vital biochemical pathways, including the beta oxidation pathway of fatty acid degradation and various biosynthetic pathways. Thiolase of *Caenorhabditis elegans* (*C. elegans*) has been demonstrated to play a key role in delaying aging of the worm and is required for lifespan extension [13,14]. It was documented that thiolase of *Homo sapiens* abolished the BNIP3 (a unique pro-apoptosis protein) induced apoptosis, which provided a possible linkage between fatty acid metabolism and apoptosis of cells [15]. Thiolases share a common evolutionary origin and a high degree of sequence similarity [16,17]. In the present study, we identified acetoacetyl-CoA thiolase of *C. sinensis* (*Cs*ACAT)*,* a homologue of thiolase. The thiolytic cleavage activity of *Cs*ACAT was successively assayed. Whether *Cs*ACAT was related to survival of the adult worm in the lipid environment of bile duct was also investigated. The collective information will be a cornerstone for learning about *Cs*ACAT and its role in *C. sinensis* survival and development in the bile duct environment containing lipid.

## Methods

### Ethics statement

Male Sprague–Dawley (SD) rats (7 weeks old) and New Zealand rabbits (2 months old) were purchased from the Animal Centre of Sun Yat-Sen University (Guangzhou, China) and raised strictly in accordance with National Institutes of Health on animal care and ethical guidelines. The experimental procedures were approved by the animal care and use committee of Sun Yat-Sen University (Permit Numbers: SCXK (Guangdong) 2009–0011).

### Parasite collection

Metacercariae of *C. sinensis* were isolated from experimentally infected freshwater fish *Pseudorasbora parva* in our ecological pool as previously described [18]. Each rat was orally infected with 50 metacercariae. 8 weeks after infection, the rats were sacrificed and *C. sinensis* adults were recovered from the livers.

### Sequence analysis of *Cs*ACAT

The full-length complementary DNA (cDNA) of *Cs*ACAT (GenBank accession No. GAA52468.1) was screened from the genome and transcriptome database of *C. sinensis* [11] by using BLAST program provided by The National Center for Biotechnology Information (http://www.ncbi.nlm.nih.gov/). InterPro-Scan and Swiss-Model provided by Expert Protein Analysis System (http://www.expasy.org/tools/) were used to analyze the conserved domains and spatial structure of *Cs*ACAT [19]. Deduced amino acid sequences alignment and dendrogram analysis of *Cs*ACAT with homologues from other species were carried out by using the software of Vector NTI.

### Quantitative real-time PCR (Q-PCR)

To investigate mRNA levels of *Cs*ACAT at different developmental stages of *C. sinensis*, total RNA was extracted from adult worms, metacercariae or eggs using TRIZOL reagent (Invitrogen, USA) according to the manufacturer’s instructions. cDNA synthesis was performed with First Strand cDNA Synthesis Kit (Fermentas, Canada) and oligo (dT)_18_ primer by using 2 μg of total RNA. Relative mRNA levels were quantified on a BIORAD iQ5 instrument (BioRad, USA) by using 100 ng of cDNA with the SYBR Premix ExTaq Kit (TaKaRa, Japan) according to the recommendations. 5′-CGTCATCTGTGCGGGCGGTAT-3′ and 5′-TTGGCGTAGGCGTCCTGCTCT-3′ were employed to amplify specific fragments of *Cs*ACAT. β-actin from *C. sinensis* (*Cs* β-actin, GenBank accession No. EU109284) was used as an internal control (323 bp) [20]. The forward and reverse primers were 5′-ACCGTGAGAAGATGACGCAGA-3′ and 5′-GCCAAGTCCAAACGAAGAATT-3′. The real-time PCR procedure was as follows: 95°C for 30 s, 40 cycles of 95°C for 5 s, and 60°C for 20 s. The melting curves were analyzed automatically by collection of the fluorescence signals. Bio-Rad iQ5 software was used to analyze the data according to 2^−ΔΔCt^ method [21].

### Preparation of antiserum of excretory/ secretory proteins from *C. sinensis* (*Cs*ESPs) and *Cs*ACAT

*Cs*ESPs were collected from isolated adult worms according to the method previously described [22].

Forward primer 5′-GTC*GGATCC*ATGGGTGGTCTGAGT-3′ with restriction site of *BamH* I (italic) and reverse primer 5′-CTC*CTCGAG*TTACCCAACCGGATGAC-3′ with restriction site of *Xho* I (italic) were employed to amplify open reading frame (ORF) of *Cs*ACAT by PCR. The amplification condition was 94°C for 30 s, 58°C for 45 s and 72°C for 1 min for 30 cycles, plus 72°C for 10 min. Specific PCR products were purified, digested with *BamH* I and *Xho* I, and ligated into pET-28a (+) plasmid digested with the same endonuclease. The recombinant plasmid pET-28a (+)-*Cs*ACAT were confirmed by DNA sequencing and transformed into *E. coli*, BL21 (DE3). *E. coli* BL21 cells harboring the recombinant plasmid were inoculated in 6 L of Luria-Bertani broth medium containing 50 μg/ml of kanamycin and cultured for about 3 h. The expression of *Cs*ACAT was induced with 0.5 mM isopropyl-β-D-thiogalactopyranoside (IPTG) for 4 h at 30°C. Recombinant *Cs*ACAT (r*Cs*ACAT) was purified with His-Bind Purification kit (Novagen, USA), and then analyzed using 12% sodium dodecyl sulfate polyacrylamide gel electrophoresis (SDS-PAGE) followed by Coomassie blue staining.

The concentration of r*Cs*ACAT or *Cs*ESPs was determined by bicinchoninic acid assay (BCA, Novagen, USA). 200 μg of r*Cs*ACAT or *Cs*ESPs were emulsified with an equal volume of complete Freund’s adjuvant and injected subcutaneously to each SD rat. Each rat was given 100 μg of protein, emulsified with equivalent incomplete Freund’s adjuvant, for three booster injections at 2-week intervals [23]. Anti-serum was collected every 2 weeks. Sera from naïve rats were also collected to use as a control.

### Immunohistochemical localization of *Cs*ACAT in *C. sinensis*

Newly isolated *C. sinensis* adults were fixed with 4% formaldehyde, embedded in paraffin wax and sliced into 3–5 μm-thick sections using a microtome (Microm, Germany). The sections were then dewaxed and dehydrated. The slides were blocked with goat serum overnight at 4°C and incubated with rat anti-r*Cs*ACAT sera (1:400 dilutions) at room temperature for 2 h. Sera from naïve rats (1:400 dilutions) were used as a negative control. The slides were washed with phosphate buffered saline (PBS) containing 0.1% Tween-20 (PBST) three times and then incubated with goat anti-rat IgG labeled with red fluorescent Cyanine dye 3 (1:400 dilutions, Cy3, Proteintech, USA) at room temperature for 1 h in the dark. 0.1% bovine serum albumin (BSA) in PBST was used as dilution buffer. After washing with PBST, fluorescence microscopy (ZEISS, Goettingen, Germany) was used in the visualization of antibody staining.

### Culture of *C. sinensis* adults with gradient lecithin

Adult worms newly recovered from infected rats were washed with sterilized PBS (pH 7.4) containing antibiotics (100 μg/ml of penicillin and 100 U/ml of streptomycin). The worms were then incubated in different concentrations of lecithin (L-α-Phosphatidylcholine, Sigma-P5394, Sigma, USA). Lecithin was firstly dissolved with 100% alcohol in a water bath maintained at 56°C and diluted with PBS to prepare the culture medium with final concentrations of lecithin of 6 mmol/L, 0.6 mmol/L and 0.06 mmol/L. The final concentration of alcohol was the same for all concentrations of culture medium i.e. 0.6%. 0.6% alcohol in PBS was used as a negative control. Finally, the mixture was homogenized using an ultrasonic instrument (285 W, 180 s, Scientz IID, China). 10 adult worms were incubated in a single well of a 6-well flat-bottom plate (Corning, USA), with 3 ml of the mixture (which was replaced every 24 hours). The worms were monitored under the microscope (Leica, Germany) for 5 min and intact live worms were counted after 12 h, 18 h, 24 h, 36 h, 48 h, and 72 h respectively. Worms with no muscle contraction or no movement after consecutive 5 shoots was recorded as dead [3,24]. All of the independent experiments were repeated in triplicate. Living adult worms were then respectively washed with 50 mM Tris–HCl (pH 7.0, buffer A) and kept in TRIZOL for total RNA extraction or sonicated in 2 ml of buffer A and then centrifuged at 12,000 g for 15 min. The supernatants were kept at 4°C for determination of protein concentration, activity assay and western blotting analysis.

### Enzymatic activity assay of r*Cs*ACAT

The thiolytic assay of r*Cs*ACAT was performed using the method previously described [25,26]. The standard reaction system contained 50 mM Tris–HCl, 20 mM MgCl_2_, 60 μM CoA (C4282, SIGMA, USA), 10 μM acetoacetyl-CoA (A1625, SIGMA, USA) and 40 mM KCl in a final volume of 0.3 mL [27]. The changes of absorption value at 303 nm were detected 10 min after addition of 0.1 μg of r*Cs*ACAT in the presence of KCl. The optimal pH and temperature of thiolytic reaction of r*Cs*ACAT were determined. The enzymatic activity of naïve ACAT in total worm extracts was measured in the optimal pH and temperature.

### Western blotting analysis

r*Cs*ACAT (5 μg), total worm extracts (50 μg), ESPs (30 μg) were subjected to 12% SDS-PAGE and then electro-transferred onto polyvinylidene difluoride (PVDF) membrane (Whatman, UK) at 100 V for 1 h in a Trans-Blot transfer cell (BIO-RAD, USA). The membranes were blocked with 5% (w/v) skimmed milk in PBS for 2 h at room temperature and then probed with mouse anti-His tag monoclonal antibody (Novagen, USA) at 1:2,000 dilutions, rat anti-r*Cs*ACAT serum (1:200), rat anti-*Cs*ESPs serum (1:200) or serum from *C. sinensis*-infected rat (1:200) at 4°C overnight, respectively. After washing with PBS, the membranes were successively incubated with HRP-conjugated rabbit anti-rat or rabbit anti-mouse IgG at 1:2000 dilutions (Proteintech Group, Chicago, USA) at room temperature for 1 h. The blots were visualized by enhanced chemiluminescence (ECL) method.

### Hyperlipidemic diet animal models with infection of *C. sinensis*

New Zealand rabbits (male) were randomly divided into two groups, a hyperlipidemic diet group and a basal diet group. Hyperlipidemic diet was composed of chow diet (78.8% w/w), pig oil (10% w/w), powdered egg yolk (10% w/w), cholesterol (1% w/w) and bile salt (0.2% w/w) [28]. Lipid levels in their sera were measured 1 month after the treatment. The concentrations of total cholesterol (TC), triglycerides (TG), high density lipoprotein (HDL) and low density lipoprotein (LDL) were measured by enzymatic methods with automated analyzer (Hitachi 7180, Hitachi, Japan). When the rabbits were confirmed as hyperlipidemic, they were intragastrically infected with 150 metacercariae. One and a half months after the infection, the rabbits were sacrificed and *C. sinensis* adults were recovered from the infected livers. The worms were prepared for detection of mRNA and protein levels of *Cs*ACAT by using quantitative real-time PCR and western blotting mentioned above. The total proteins of adult worms from each group were subjected to western blotting analysis in the same quantity as a rigorous control. In addition, serum samples were taken when rabbits were starved for 2 hours. Lipids levels in serum and bile from each rabbit were measured.

### Statistical analysis

Statistical analysis was performed with SPSS 13.0 Software (SPSS, München, Germany) using either Student *t*-test or one-way ANOVA when appropriate. Results represented means ± s.d. of triplicates per experimental condition. *p*-value <0.05 was considered as significant.

## Results

### Sequence analysis of *Cs*ACAT

The ORF of *Cs*ACAT encoding 284 amino acids contained highly conserved motifs of thiolase-like region. The thiolase folds or representative functional domain of the thiolase superfamily were identified in spatial structures of *Cs*ACAT. There were significant similarities among tertiary structures of ACAT from *C. sinensis*, *Homo sapiens*, and *C. elegans* (Additional file 1: Figure S1A). Multi-sequence alignment analysis indicated that the deduced amino acid sequence of *Cs*ACAT shared 56%, 55%, 55%, 52%, and 51% identity with those of *Drosophila melanogaster* (XP_002057374.1), *Homo sapiens* (BAA01387.1), *Trichinella spiralis* (XP_003380495.1), *Ascaris suum* (ADY43074.1), and *C. elegans* (NP_495455.2). A phylogenetic analysis showed that *Cs*ACAT was homologous with groups of trematodes such as *S. japonicum* and nematodes such as *C. elegans* (Additional file 1: Figure S1B). The mammalian ACAT formed a major clade with ACAT from bird and fish. The insect ACAT grouped a clade from others.

### Expression, purification, and identification of *Cs*ACAT

**Figure 1:**
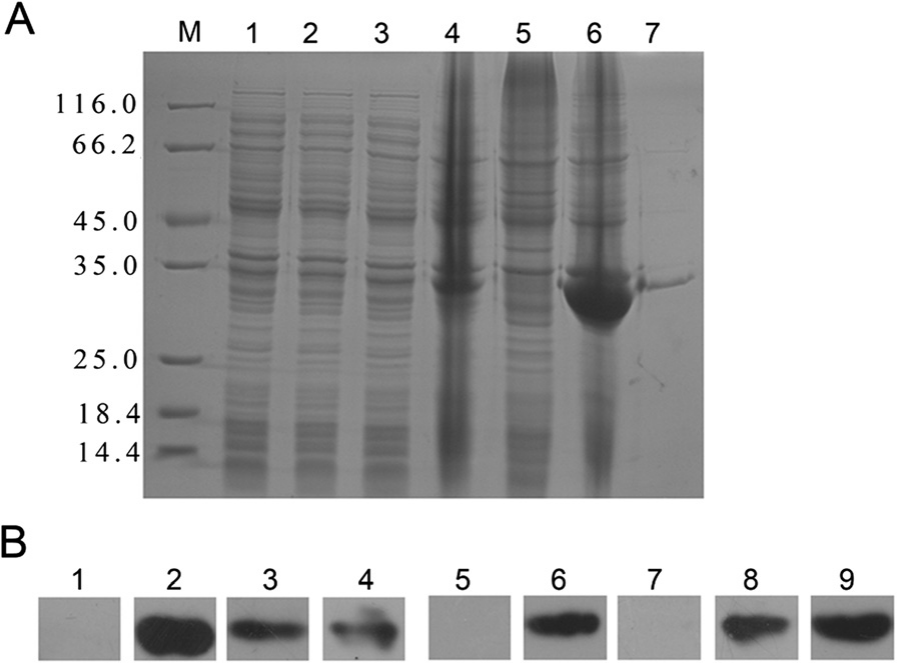
**Expression, purification and characterization of** ***Cs*** **ACAT. (A)** r*Cs*ACAT was identified by 12% SDS-PAGE. Protein molecular weight markers (*lane M*), lysate of *E. coli* containing pET28a (+) without induction (*lane 1*) and with induction by IPTG (*lane 2*), lysate of *E. coli* containing pET28a (+)-*Cs*ACAT without induction (*lane 3*) and with induction by IPTG (*lane 4*), supernatant (*lane 5*) and precipitant (*lane 6*) of lysate of *E. coli* containing the recombinant plasmid after induction, the purified recombinant *Cs*ACAT protein (*lane 7*). **(B)** Western blotting analysis of *Cs*ACAT. *Lane 1–4*, r*Cs*ACAT probed with naive serum, anti-His tag monoclonal antibody, rat anti-r*Cs*ACAT serum and serum from *C. sinensis*-infected rat; *lane 5–6*, *Cs*ESPs probed with naive serum and rat anti-r*Cs*ACAT serum; *lane 7–8*, total proteins of adult worm probed with naive serum and rat anti-r*Cs*ACAT serum; *lane 9*, purified r*Cs*ACAT blotted with rat anti-*Cs*ESPs serum.

The ORF of *Cs*ACAT was cloned into pET-28a (+) expression vector and the recombinant plasmids were confirmed by sequencing. The IPTG induced r*Cs*ACAT was purified and analyzed by SDS-PAGE, showing a single band with molecular mass of approximately 30 kDa (Figure 1A). Western blotting (Figure 1B) indicated that r*Cs*ACAT could be probed by mouse anti-His tag monoclonal antibody, rat anti-*Cs*ACAT serum, rat anti-*Cs*ESPs serum, and serum from *C. sinensis*-infected rat at a clear band about 30 kDa, while r*Cs*ACAT could not be blotted with serum from pre-immunized rat. *Cs*ESPs and total proteins of worm could also be probed by rat anti-*Cs*ACAT serum but not by serum from pre-immunized rat. In addition, the optimum enzymatic activity of r*Cs*ACAT was at pH 7.5 and 37°C (Additional file 2: Figure S2).

### Expression pattern of *Cs*ACAT

**Figure 2:**
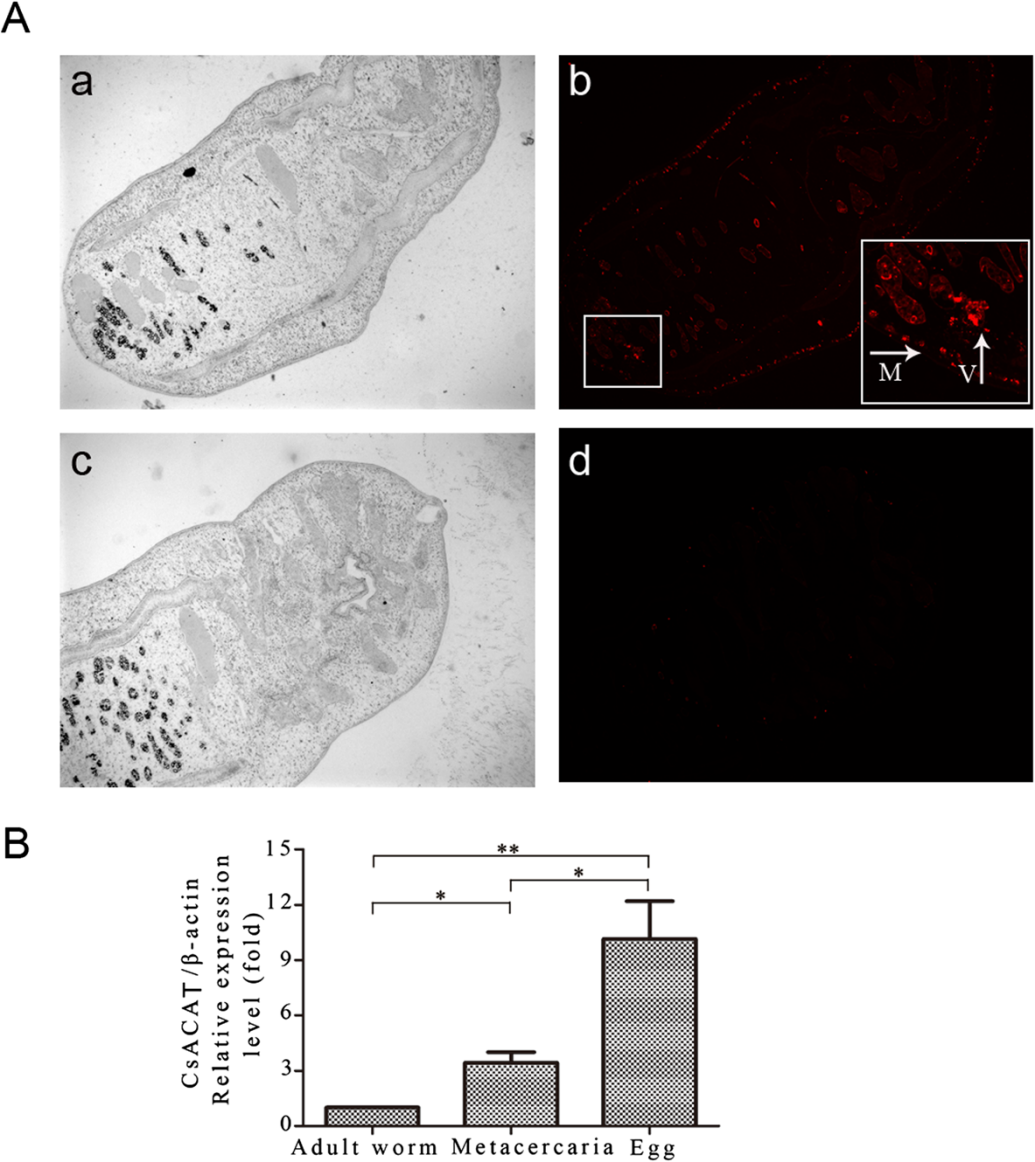
**Expression pattern of** ***Cs*** **ACAT. (A)** Immunolocalization of *Cs*ACAT in *C. sinensis* adult. Panel *a* and *b,* sections treated with anti-r*Cs*ACAT serum and specific fluorescences distributed in the vitellarium and sub-tegumental muscular layer of the adult worm; Panel *c* and *d*, sections treated with naive serum. No fluorescence was detected. *M*, sub-tegumental muscular layer; *V*, vitellarium. Magnification for the adult worm was × 100. **(B)** mRNA level of *Cs*ACAT at different developmental stages of *C. sinensis* by quantitative real-time PCR. The specific mRNA fragment of *Cs*ACAT was observed among the stages. The quantities were normalized with *Cs* β-actin and analyzed by means of the 2^−ΔΔCt^ ratio.

The specific fluorescences were specifically localized in the vitellarium and sub-tegumental muscle layer of the adult worm by using rat anti-r*Cs*ACAT serum, while no specific fluorescence was observed in sections treated with naive serum (Figure 2A). The result of quantitative real-time PCR demonstrated that mRNA of *Cs*ACAT was observed at life stages of adult worm, metacercaria and egg of *C. sinensis*. The mRNA level of *Cs*ACAT in egg was higher than that in metacercaria (2.93-fold, *p* < 0.05) or adult worm (10.16-fold, *p* < 0.01) (Figure 2B).

### Survival rate of adult worms cultured in lecithin

**Figure 3:**
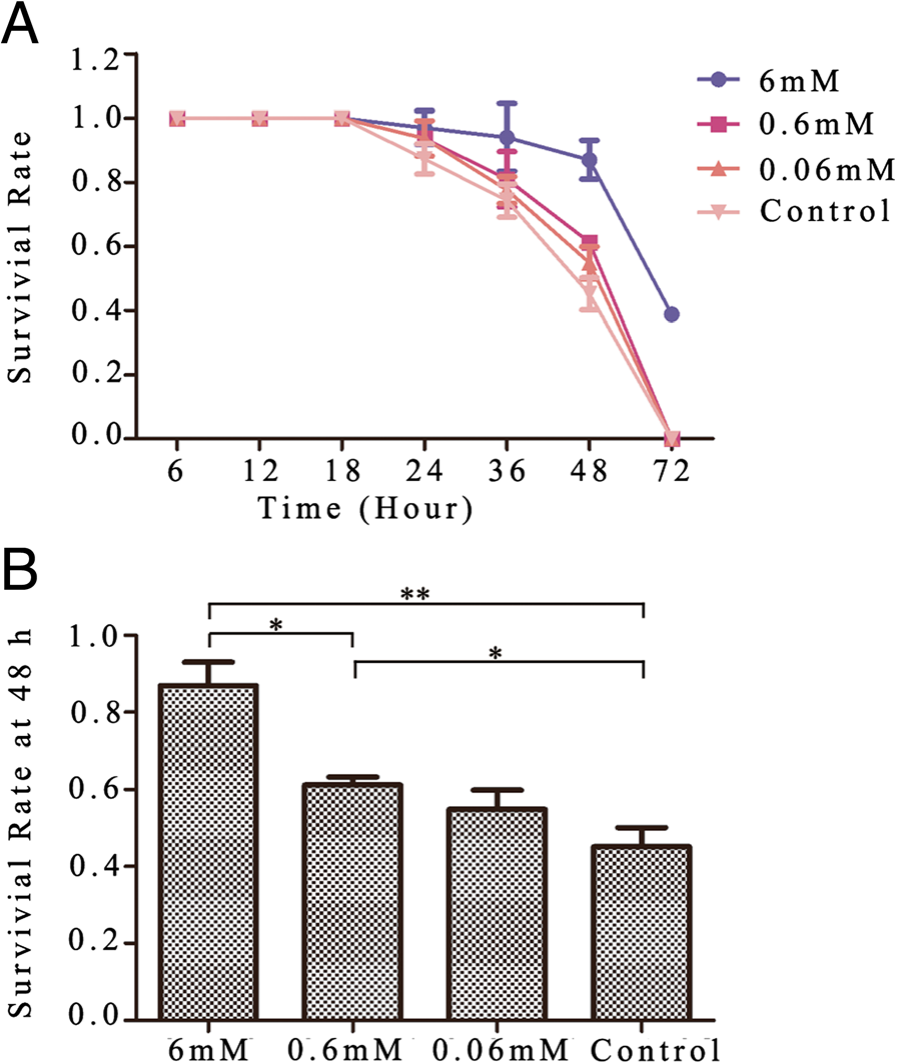
**Survival rate of adult worms cultured in lecithin. (A)** Survival rates of adult worms in all groups were decreased in a time-dependent manner. **(B)** Survival rates of worms in different concentrations of lecithin at 48 h. All data are presented as mean ± SD, *p < 0.05, **p < 0.01.

In gradient concentrations of lecithin from 6 mM to 0.06 mM, the survival rate of adult worms declined in a time-dependent manner from 18 h. The survival rate of 6 mM lecithin group was statistically the highest. The survival rates were 87.0 ± 6.0% in 6 mM, 57.9 ± 7.1% in 0.6 mM, 54.8 ± 5.0% in 0.06 mM lecithin, and 45.2 ± 5.0% in the control group at 48 h. At 72 h, the survival rates were 38.8 ± 2.0% in 6 mM lecithin, 0% in 0.6 mM, 0% in 0.06 mM lecithin and 0% in the control group (Figure 3).

### Enzymatic activity and expression level of *Cs*ACAT in adult worms incubated in lecithin

**Figure 4:**
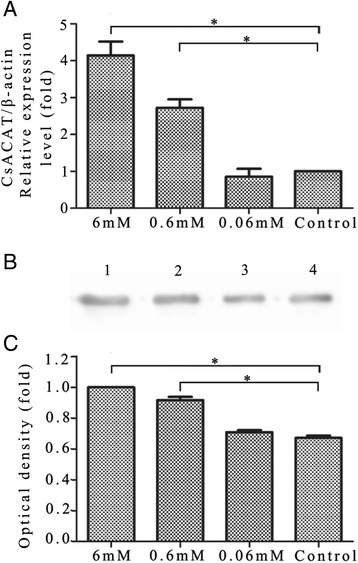
**Expression level and enzymatic activity of** ***Cs*** **ACAT in adult worms incubated in lecithin. (A)** mRNA levels of *Cs*ACAT in worms cultured with different concentrations of lecithin were evaluated by quantitative real-time PCR. **(B)** Crude proteins extracted from worms cultured in 6 mM (*Lane 1*), 0.6 mM (*Lane 2*), 0.06 mM (*Lane 3*) of lecithin and control group (*Lane 4*) were probed with rat anti-r*Cs*ACAT serum. **(C)** Optical density analysis of the protein levels. The optical density was calculated by Tanon Gis software and analyzed by Student’s *t* test, *p < 0.05.

Living worms were collected 48 h after incubation in lecithin and subjected to crude extraction to examine enzymatic activity of *Cs*ACAT. The *Cs*ACAT activity in newly recovered worms taken from rats was set as 100%. The enzymatic activity of *Cs*ACAT from worms cultured in 6 mM lecithin showed a highest activity of 40.19% compared with other groups (Table 1). Both mRNA and protein levels of *Cs*ACAT were higher in 6 mM lecithin and 0.6 mM lecithin groups compared with those of the control group by Q-PCR (Figure 4A) and Western blotting (Figure 4B-C).

**Table 1:** **Enzymatic activity of** ***Cs*** **ACAT in adult worms incubated with different concentrations of lecithin**

**Fraction**	**Concentration (μg/μl)**	**Volume (μl)**	**Total protein (μg)**	**Total activity units (μmol/min)**	**Specific activity units/mg protein**	**Relative activity (%)**
Naïve ACAT activity#	6.68	5	33.41	0.024	0.729	100
6 mM lecithin*	9.82	5	49.09	0.014	0.293	40.19
0.6 mM lecithin*	11.03	5	55.15	0.01	0.181	24.81
0.06 mM lecithin*	12.99	5	64.95	0.011	0.168	23.03
Control**	11.12	5	55.58	0.006	0.104	14.31

The crude extracts of worms incubated with different conditions were collected at 48 h. #, Enzymatic activity of *Cs*ACAT in worms newly recovered from liver of infected rat was set as 100%; *, Lecithin was firstly dissolved with 100% alcohol in a 56°C water bath and then diluted with PBS to prepare the mixture containing different concentrations of lecithin; **, Worms cultured with 0.6% alcohol in PBS was applied as a negative control.

### Expression level of *Cs*ACAT in adult worm recovered from hypercholesteremia rabbit models

**Figure 5:**
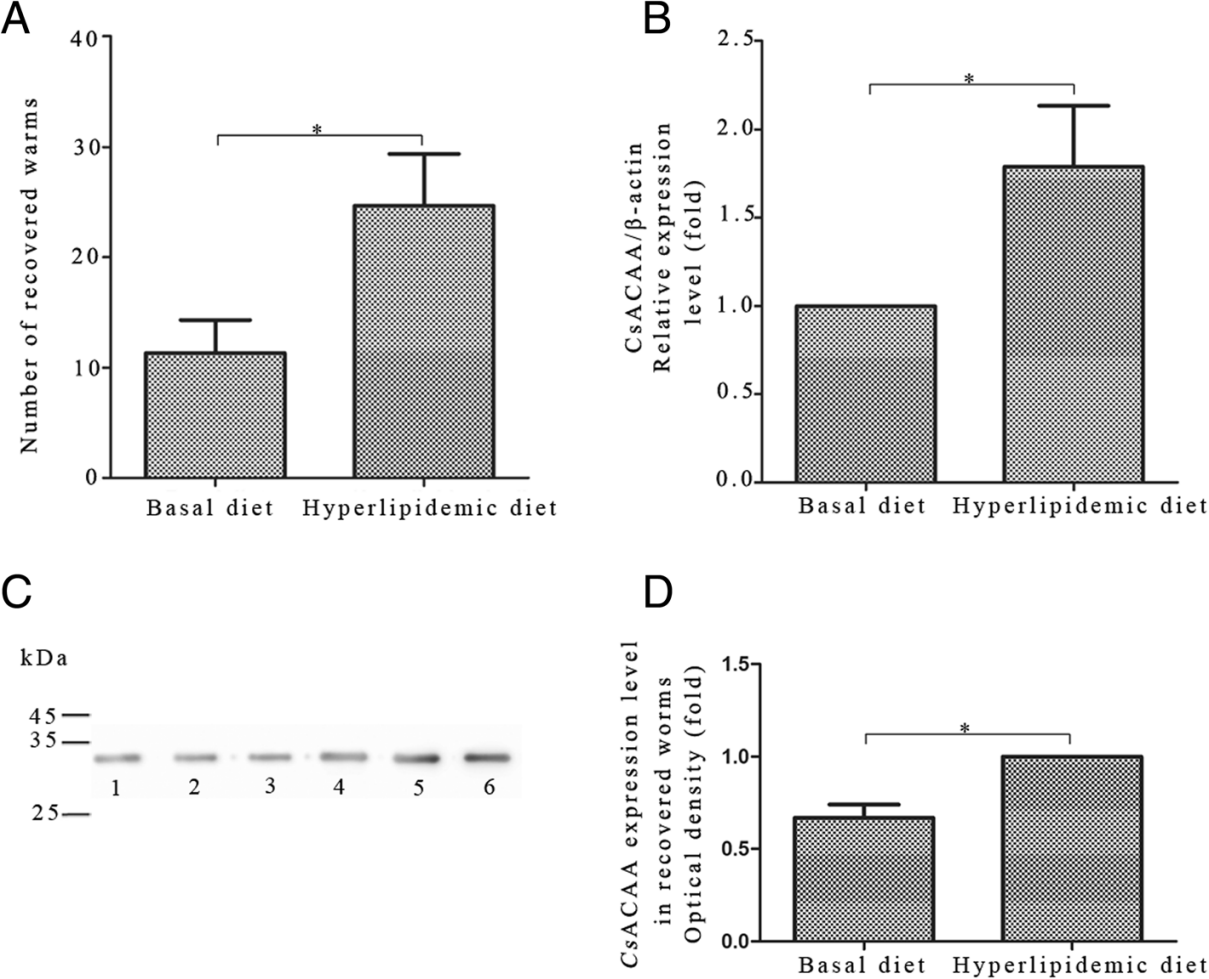
**Worm burden in rabbit models and expression level of** ***Cs*** **ACAT in the worms. (A)** Worm burden in hypercholesteremia or basal diet fed rabbits 6 weeks after *C. sinensis* infection. All data are presented as mean ± SD, *p < 0.05, **p < 0.01. **(B)** The mRNA level of *Cs*ACAT in adult worms extracted from hypercholesteremia or basal diet fed rabbits, normalized with *Cs* β-actin. **(C)** The protein levels of *Cs*ACAT in adult worms of the two groups. *Lane 1–3*, total proteins of worms recovered from basal diet treated rabbits probed with rat anti-r*Cs*ACAT serum. *Lane 4–6*, total proteins of worms recovered from hypercholesteroemic rabbits blotted with rat anti-r*Cs*ACAT serum. **(D)** Optical density analysis of the protein levels in Figure 5C. The optical density was calculated by Tanon Gis software and analyzed by Student’s *t* test, *p < 0.05.

The total cholesterol (TC) levels in sera from rabbits fed a hyperlipidemic diet were much higher than those of the basal diet group after 30 days (Additional file 3: Figure S3A). It verified the successful construction of hypercholesteremia rabbit models. 6 weeks after infection with *C. sinensis*, TC levels in sera and bile collected from the hypercholesteremia rabbits were much higher than those of rabbits fed with basal diet. TG levels showed no statistical difference in the sera and bile between the two groups (Additional file 3: Figure S3B-C). More worms were recovered from livers of hypercholesteremia rabbits than from the basal diet group (Figure 5A). The mRNA and protein levels of *Cs*ACAT were higher in the worms extracted from hypercholesterolemic rabbits than those of the control group (Figure 5B-D).

## Discussion

ACAT is a member of the thiolases superfamily. Thiolases function in both degradative and synthetic pathways of fatty acids. In the degradative direction, ACAT can catalyze the thiolytic cleavage of acetoacetyl-CoA molecules into two molecules of acetyl-CoA [26]. The optimal thiolytic activity of r*Cs*ACAT to cleave acetoacetyl-CoA was at pH 7.5 and 37°C (Additional file 2: Figure S2) and native *Cs*ACAT had thiolytic activity too (Table 1), confirming that *Cs*ACAT is a member of the thiolase family.

ESPs released by *C. sinensis* adults are complex, including proteases, antioxidant enzymes and metabolic enzymes, which play important roles in the reaction between the parasite and the host [22,29]. The crude extracts of worms and *Cs*ESPs could be blotted with rat anti-r*Cs*ACAT sera at a specifically single band. r*Cs*ACAT could be probed with rat anti-*Cs*ESPs and sera from infected rats. The results indicated that *Cs*ACAT was an ingredient of *Cs*ESPs as well as a circulating antigen. *Cs*ACAT was specifically localized at the vitellarium and sub-tegumental muscle layer in adult worms by immunofluorescence. The localizations have close relationships with lipid metabolism and energy consumption. The mRNA level of *Cs*ACAT in egg was much higher than those in adult worms and metacercariae. Fatty acid catabolism was documented to be essential for egg production in *schistosomes* [30] and might also be of importance in *C. sinensis*. Collectively, it was the first report on the molecular characterizations of *Cs*ACAT, which was a cornerstone for further investigation of its role in physiology of *C. sinensis.*

*C. sinensis* adults survive in a bile environment which contains lipids (lecithin, cholesterol and fatty acids). As mentioned, mapped to Kyoto Encyclopedia of Genes and Genomes (KEGG) pathways of gene models, enzymes functioning in fatty acid metabolism were completely intact and expressed in *C. sinensis* while not in *S. japonicum* [11,12]. Enzymatic activity of these enzymes and their roles in physiology and parasitism of *C. sinensis* were worth clarifying. We firstly investigated the influences of different levels of lipids on *Cs*ACAT and the fluke *in vivo* and *in vitro*.

Lecithin and cholesterol are the major lipids in human bile. The concentrations of lecithin and cholesterol in human bile are 4.1-9.3 mM [31] and 1.6-8.3 mM [32], respectively. On the one hand, it was difficult to imitate the physical concentration of cholesterol due to solidification of the solution at 37°C *in vitro*, therefore, adult worms were cultured in gradient concentrations of lecithin from 0.06 mM to 6 mM. On the other hand, *in vivo* lecithin could be converted into free fatty acid by lyso-phospholipases of *C. sinensis,* which have previously been confirmed to be components of *Cs*ESPs and to be responsible for the activity [33,34]. It might be a way to supply free fatty acids for *C. sinensis* as materials for beta-oxidation in the bile environment after transportation by the fatty acid binding protein (FABP) [30,34]. As a result, the survival rate of adult worms in 6 mM lecithin was statistically higher than those in 0.6 mM, 0.06 mM lecithin and controls at 48 h. Simultaneously, the enzymatic activity of *Cs*ACAT to convert acetoacetyl-CoA molecules into two molecules of acetyl-CoA in worms cultured in 6 mM lecithin was the highest in the four groups, as well as the expression level of *Cs*ACAT. Moreover, more worms were recovered from hypercholesteremia animal models with higher TC levels in the bile. The expression level of *Cs*ACAT in the worms was also higher than that of the basal diet group. The results *in vivo* and *in vitro* implied that *C. sinensis* might sense lipid levels and survive better in the bile environment with higher lipid levels. The findings suggested that hypercholesteremia might increase the risk of infections of *C. sinensis*.

*In vitro*, lecithin in mixed micelles attenuates the cytotoxicity of bile salts on Caco-2 cells [4]. It has been documented that abundant fat oxidation could delay aging of *C. elegans* [35]. In *C. elegans*, function loss of thiolase gene kat-1 causes accelerated aging. The effect of kat-1 might be a consequence of an influence on fatty acid oxidation [13,14,36,37]. *Cs*ACAT converts acetoacetyl-CoA molecules into two molecules of acetyl-CoA and the latter may enter into the tricarboxylic acid cycle (TCA cycle) for energy production. Our transcriptome [11] and unpublished data showed that the enzymes involved in the TCA cycle intactly expressed in the adult stage. *C. sinensis* might utilize lipids by up-regulating expression and enzymatic activity of *Cs*ACAT to enhance beta oxidation pathways of fatty acid as an energy resource to survive longer, which might contribute to the higher survival rate of adult worms in an environment with a higher level of lipids.

Collectively, in the present study, we identified and characterized *Cs*ACAT, and investigated its potential role in survival of the worm in the bile duct. The expression level and enzymatic activity of *Cs*ACAT were up-regulated with the enhancement of lipid concentration *in vivo* and *in vitro*. Simultaneously, the survival rates of adult worms were increased. Our findings suggested that as a parasitic organism in a bile environment, *C. sinensis* might modulate the expression and enzymatic activity of *Cs*ACAT, an enzyme involved in fatty acid metabolism, for energy or physical requirements to efficiently adapt to surviving in the bile duct of the host.

## Conclusions

In summary, our results implied that *C. sinensis* might sense lipid levels and survive better in the bile environment with higher lipid levels. *Cs*ACAT, an enzyme involved in fatty acid metabolism, might play an important role in the efficient adaption of *C. sinensis* to the bile environment with lipids in host.

## Additional files

Additional file 1: Figure S1. Putative tertiary modeling and phylogram tree analysis of *Cs*ACAT. **(A)** Tertiary structure of *Cs*ACAT. There were significant similarities among tertiary structures of ACAT from *C. sinensis*, *Homo sapiens*, and *C. elegans*. **(B)** The phylogram tree analysis of *Cs*ACAT. The sequences included were as follows: *Anas platyrhynchos* (EOA96764.1), *Columba livia* (EMC80495.1), *Homo sapiens* (BAA01387.1), *Pongo abelii* (XP_002822478.1), *Macaca mulatta* (NP_001252622.1), *Felis catus* (XP_003992377.1), *Mus musculus* (BAE37335.1), *Rattus norvegicus* (NP_058771.2), *Maylandia zebra* (XP_004561662.1), *Xiphophorus maculatus* (XP_005806760.1), *Osmerus mordax* (ACO09524.1), *Ascaris suum* (ADY43074.1), *Caenorhabditis elegans* (NP_495455.2), *Drosophila mojavensis* (XP_002009553.1), *Drosophila persimilis* (XP_002022751.1), *Nasonia vitripennis* (XP_001607694.1), *Tribolium castaneum* (XP_975008.2) and *Schistosoma japonicum* (AAX26758.2). Additional file 2: Figure S2. Enzymatic activity of r*Cs*ACAT at different pH and temperature using acetoacetyl-CoA as the substrate. **(A)** Enzymatic activity of r*Cs*ACAT at pH 6.0-9.5. **(B)** Enzymatic activity of r*Cs*ACAT at 10–65°C. Additional file 3: Figure S3. Concentrations of total cholesterol (TC), triglycerides (TG), high density lipoprotein (HDL) and low density lipoprotein (LDL) in sera or bile collected from rabbits treated with hyperlipidemic or basal diet. Levels of TC, TG, LDL and HDL in sera of treated rabbits after 1 month **(A)** and 2.5 months **(B)**. TC and TG levels in bile of treated rabbits after 2.5 months **(C)**. All data are presented as mean ± SD, **p* < 0.05, ***p* < 0.01.

## References

[1] Wu W, Qian X, Huang Y, Hong Q (2012). A review of the control of *clonorchiasis sinensis* and *Taenia solium* taeniasis/cysticercosis in China. Parasitol Res.

[2] Hong ST, Fang Y (2012). *Clonorchis sinensis* and clonorchiasis, an update. Parasitol Int.

[3] Uddin MH, Li S, Bae YM, Choi MH, Hong ST (2012). In vitro maintenance of *clonorchis sinensis* adult worms. Korean J Parasitol.

[4] Tan Y, Qi J, Lu Y, Hu F, Yin Z, Wu W (2013). Lecithin in mixed micelles attenuates the cytotoxicity of bile salts in Caco-2 cells. Toxicol In Vitro.

[5] Lim MK, Ju YH, Franceschi S, Oh JK, Kong HJ, Hwang SS (2006). *Clonorchis sinensis* infection and increasing risk of cholangiocarcinoma in the Republic of Korea. Am J Trop Med Hyg.

[6] Legido-Quigley C (2010). Metabolite-biomarker investigations in the life cycle of and infection with *Schistosoma*. Parasitology.

[7] Kita K, Nihei C, Tomitsuka E (2003). Parasite mitochondria as drug target: diversity and dynamic changes during the life cycle. Curr Med Chem.

[8] Iwata F, Shinjyo N, Amino H, Sakamoto K, Islam MK, Tsuji N (2008). Change of subunit composition of mitochondrial complex II (succinate-ubiquinone reductase/quinol-fumarate reductase) in *Ascaris suum* during the migration in the experimental host. Parasitol Int.

[9] van Erpecum KJ (2005). Biliary lipids, water and cholesterol gallstones. Biol Cell.

[10] Soderlind E, Karlsson E, Carlsson A, Kong R, Lenz A, Lindborg S (2010). Simulating fasted human intestinal fluids: understanding the roles of lecithin and bile acids. Mol Pharm.

[11] Wang X, Chen W, Huang Y, Sun J, Men J, Liu H (2011). The draft genome of the carcinogenic human liver fluke *Clonorchis sinensis*. Genome Biol.

[12] Zhou Y, Zheng H, Chen Y, Zhang L, Wang K (2009). The *Schistosoma japonicum* genome reveals features of host-parasite interplay. Nature.

[13] Wang MC, O’Rourke EJ, Ruvkun G (2008). Fat metabolism links germline stem cells and longevity in C. elegans. Science.

[14] Berdichevsky A, Nedelcu S, Boulias K, Bishop NA, Guarente L, Horvitz HR (2010). 3-Ketoacyl thiolase delays aging of *Caenorhabditis elegans* and is required for lifespan extension mediated by sir-2.1. Proc Natl Acad Sci U S A.

[15] Cao W, Liu N, Tang S, Bao L, Shen L, Yuan H (2008). Acetyl-Coenzyme A acyltransferase 2 attenuates the apoptotic effects of BNIP3 in two human cell lines. Biochim Biophys Acta.

[16] Igual JC, Gonzalez-Bosch C, Dopazo J, Perez-Ortin JE (1992). Phylogenetic analysis of the thiolase family. Implications for the evolutionary origin of peroxisomes. J Mol Evol.

[17] Bun-Ya M, Maebuchi M, Hashimoto T, Yokota S, Kamiryo T (1997). A second isoform of 3-ketoacyl-CoA thiolase found in *Caenorhabditis elegans*, which is similar to sterol carrier protein x but lacks the sequence of sterol carrier protein 2. Eur J Biochem.

[18] Liang C, Hu XC, Lv ZY, Wu ZD, Yu XB, Xu J (2009). Experimental establishment of life cycle of *Clonorchis sinensis*. Zhongguo Ji Sheng Chong Xue Yu Ji Sheng Chong Bing Za Zhi.

[19] Xu Y, Chen W, Bian M, Wang X, Sun J, Sun H (2013). Molecular characterization and immune modulation properties of *Clonorchis sinensis*-derived RNASET2. Parasit Vectors.

[20] Yoo WG, Kim TI, Li S, Kwon OS, Cho PY, Kim TS (2009). Reference genes for quantitative analysis on *Clonorchis sinensis* gene expression by real-time PCR. Parasitol Res.

[21] Livak KJ, Schmittgen TD (2001). Analysis of relative gene expression data using real-time quantitative PCR and the 2 (−Delta Delta C(T)) Method. Methods.

[22] Kim TI, Na BK, Hong SJ (2009). Functional genes and proteins of *Clonorchis sinensis*. Korean J Parasitol.

[23] Liang P, Zhang F, Chen W, Hu X, Huang Y, Li S (2013). Identification and biochemical characterization of adenylate kinase 1 from *Clonorchis sinensis*. Parasitol Res.

[24] Huang C, Xiong C, Kornfeld K (2004). Measurements of age-related changes of physiological processes that predict lifespan of *Caenorhabditis elegans*. Proc Natl Acad Sci U S A.

[25] Middleton B (1973). The oxoacyl-coenzyme a thiolases of animal tissues. Biochem J.

[26] Haapalainen AM, Merilainen G, Pirila PL, Kondo N, Fukao T, Wierenga RK (2007). Crystallographic and kinetic studies of human mitochondrial acetoacetyl-CoA thiolase: the importance of potassium and chloride ions for its structure and function. Biochemistry.

[27] Santhanam S, Venkatraman A, Ramakrishna BS (2007). Impairment of mitochondrial acetoacetyl CoA thiolase activity in the colonic mucosa of patients with ulcerative colitis. Gut.

[28] Hou Y, Shao W, Xiao R, Xu K, Ma Z, Johnstone BH (2009). Pu-erh tea aqueous extracts lower atherosclerotic risk factors in a rat hyperlipidemia model. Exp Gerontol.

[29] Zheng M, Hu K, Liu W, Li H, Chen J, Yu X (2013). Proteomic analysis of different period excretory secretory products from *Clonorchis sinensis* adult worms: molecular characterization, immunolocalization, and serological reactivity of two excretory secretory antigens-methionine aminopeptidase 2 and acid phosphatase. Parasitol Res.

[30] Huang L, Hu Y, Huang Y, Fang H, Li R, Hu D (2012). Gene/protein expression level, immunolocalization and binding characteristics of fatty acid binding protein from *Clonorchis sinensis* (*Cs*FABP). Mol Cell Biochem.

[31] Balint JA, Kyriakides EC, Spitzer HL, Morrison ES (1965). Lecithin fatty acid composition in bile and plasma of Man, dogs, rats, and oxen. J Lipid Res.

[32] Tso P, Rhoades RA, Bell DR (2012). Gastrointestinal secretion, digestion, and absorption. Medical Physiology: Principles of Clinical Medicine (MEDICAL PHYSIOLOGY (RHOADES)).

[33] Ma C, Hu X, Hu F, Li Y, Chen X, Zhou Z (2007). Molecular characterization and serodiagnosis analysis of a novel lysophospholipase from *Clonorchis sinensis*. Parasitol Res.

[34] Zhang F, Liang P, Chen W, Wang X, Hu Y, Liang C (2013). Stage-specific expression, immunolocalization of *Clonorchis sinensis* lysophospholipase and its potential role in hepatic fibrosis. Parasitol Res.

[35] Bargmann CI, Horvitz HR (1991). Control of larval development by chemosensory neurons in *Caenorhabditis elegans*. Science.

[36] Mak HY, Nelson LS, Basson M, Johnson CD, Ruvkun G (2006). Polygenic control of *Caenorhabditis elegans* fat storage. Nat Genet.

[37] Sugawara S, Honma T, Ito J, Kijima R, Tsuduki T (2013). Fish oil changes the lifespan of *Caenorhabditis elegans* via lipid peroxidation. J Clin Biochem Nutr.

